# Psychopathological symptom severity and parent–child age differences in patients with eating disorders

**DOI:** 10.1007/s40211-025-00528-4

**Published:** 2025-06-16

**Authors:** Lina Schmauch, Emma Josephine Michel, Günter Reich, Thomas Meyer

**Affiliations:** Department of Psychosomatic Medicine and Psychotherapy, University Medical Center Göttingen, Von-Siebold-Str. 5, Göttingen, Germany

**Keywords:** Anorexia nervosa, Bulimia nervosa, Mother–patient age difference, Father–patient age difference, Parent–child age gap, Anorexia nervosa, Bulimia nervosa, Mutter-Patienten-Altersunterschied, Vater-Patienten-Altersunterschied, Eltern-Kind-Altersabstand

## Abstract

**Background:**

Although family factors affecting the development of eating disorders (EDs) have been extensively studied, it is unknown whether the age gaps between the parents and their affected child have an impact on the overall psychopathological symptom severity.

**Methods:**

In a cohort of 510 consecutive study participants diagnosed with an ED and treated between 1991 and 2017, we examined the associations between parent–patient age gaps and scores from the self-report Global Severity Index (GSI), which measures overall psychopathological distress and symptom severity of the nine psychopathological themes of the Symptom Checklist-90-Revised (SCL-90-R).

**Results:**

In univariate analysis, the GSI scores correlated significantly and negatively between the ED-affected child and the age gap to both the mother (r = −0.119, *p* = 0.007) and the father (r = −0.108, *p* = 0.017). Regression models adjusted for body mass index, living in the parental home, and the education level of the patient as clinically relevant confounders confirmed the significant relationship between the overall psychological distress and the age difference to the mother (exp(β) = −0.091, 95% confidence interval [CI] = −0.022–0.000, *p* = 0.049). In addition, using the same set of confounders, there was a trend toward an association between GSI scores and the paternal age difference to the ED-diagnosed patients (exp(β) = −0.086, 95% CI = −0.018–0.000, *p* = 0.063).

**Conclusion:**

The symptom severity is more pronounced in cases of a smaller age gap between the parents and their ED-affected child. In multivariate analysis, a significant association was found between a higher self-rated symptom severity and a smaller maternal age difference to the patient. Future studies should thoroughly investigate the influence of parents’ age on their offspring’s EDs in search of potential underlying mechanisms.

## Abstract (de)

**Hintergrund:**

Obwohl familiäre Faktoren, die sich auf die Entwicklung von Essstörungen (ED) auswirken, bereits eingehend untersucht wurden, ist nicht bekannt, ob Altersunterschiede zwischen Eltern und betroffenem Kind Einfluss auf die Intensität der psychopathologischen Symptomatik haben.

**Methoden:**

In einer Kohorte von 510 konsekutiven Studienteilnehmern, die zwischen 1991 und 2017 mit einer ED diagnostiziert und therapiert wurden, untersuchten wir Assoziationen zwischen dem Eltern-Patienten-Altersabstand und den Scores im selbstberichteten Global Severity Index (GSI), der den psychopathologischen Stress insgesamt und die Symptomschwere der 9 psychopathologischen Skalen der SCL-90‑R (Symptom Checklist-90-Revised) erfasst.

**Ergebnisse:**

In der univariaten Analyse unterschieden sich die GSI-Scores signifikant und negativ zwischen dem ED-betroffenen Kind und dem Altersabstand sowohl zur Mutter (r = –0,119, *p* = 0,007) als auch zum Vater (r = –0,108, *p* = 0,017). Um Body-Mass-Index, Wohnen im Elternhaus und Bildungsniveau der Patientin als klinisch relevante Confounder korrigierte Regressionsmodelle bestätigten die signifikante Beziehung zwischen der psychischen Gesamtbelastung und dem Altersunterschied zur Mutter (exp(β) = –0,091, 95 %-Konfidenzinterval [KI] = –0,022–0,000, *p* = 0,049). Darüber hinaus gab es unter Verwendung derselben Gruppe von Confoundern einen Trend hin zu einer Assoziation zwischen dem GSI-Score und dem Altersunterschied zwischen den Vätern und den Patientinnen mit der ED-Diagnose (exp(β) = –0,086, 95 %-KI = –0,018–0,000, *p* = 0,063).

**Schlussfolgerung:**

Die Symptombelastung ist bei einem geringeren Altersunterschied zwischen den Eltern und ihrem von einer ED betroffenen Kind stärker ausgeprägt. In der multivariaten Analyse wurde ein signifikanter Zusammenhang zwischen einer höheren selbstbeurteilten Symptombelastung und einem geringeren mütterlichen Altersabstand zu den Patientinnen festgestellt. In künftigen Studien sollte der Einfluss des Alters der Eltern auf die ED ihrer Nachkommen eingehend untersucht werden, um mögliche zugrunde liegende Mechanismen zu erfassen.

## Introduction

Parental factors are considered to be important components in the etiology and course of eating disorders (EDs). Interaction patterns of parents can influence the eating behavior of their children in childhood, adolescence, and early adulthood [1, 2]. Several risk factors for the development of pathological eating behavior have been identified in the literature, such as the prevalent stress level in the family [3], maternal and paternal education [3], a history of EDs in the mother [3], and parental socioeconomic status [3, 4]. Given the trend towards delayed childbirth in recent years [5–7], the study of the influence of parental age on the manifestation of ED has become increasingly important.

Numerous studies have reported a relationship between parental age and prevalence of ED in the offspring [8–14]. Some investigations have shown a correlation between parental age and the risk of developing an ED, with some studies demonstrating a positive association and others coming to contradictory conclusions. In a small cohort of 40 patients with bulimia nervosa compared to normal controls, Dolan et al. reported that a significantly higher proportion of parents were of older age [15], and similarly, in a sample of 94 anorexic patients, Halmi found a higher parental age [16]. In contrast, in a large nationwide population study based on data from the Danish Psychiatric Central Research Register, no clear associations were found between maternal or paternal age and pathological eating behaviors in children [17]. More recently, using data from the FamFINED study, we reported that the maternal age at childbirth was on average higher in patients with anorectic eating than in patients diagnosed with bulimia nervosa from the same study population [18].

Studies reporting on the association between parental age at birth and ED incidence have come to conflicting conclusions. In an American cohort, higher paternal age due to demographic changes was associated with an increase in the incidence of anorexia nervosa among female participants [19]. An analysis of combined data from a nationwide Swedish population and healthcare registers found that the incidence of anorexia nervosa, bulimia nervosa and ED not otherwise specified (EDNOS) showed a positive correlation with higher paternal age at birth, even after controlling for maternal age as a covariate [14]. In a large cohort study of female twins, a positive association was found between older paternal age at birth and the presence or history of ED symptoms, with patients whose fathers were younger than 40 years of age being less likely to have EDs [12]. In contrast, Koch et al. found no independent effect of older fathers at childbirth on the development of EDs [20]. A recent population-based study from Taiwan, which examined 7 million citizens, provided evidence, demonstrating a U-shaped relationship between paternal age and the likelihood of their offspring developing an ED [21].

As shown in some clinical studies, children whose mothers were older at birth were more likely to develop symptoms of pathological eating behavior [20, 22]. It should be noted, however, that most studies examining the association between parental age and an ED have focused on AN, with less data available for other entities such as BN, binge ED or EDNOS. Recently, in a sample of 408 college students aged 18–23 years, Lofrano-Prado et al. found a lower risk of anorectic symptoms, when their mothers were 25 years or older at birth [23]. In addition, it is important to add that the association between ED and parental age at birth could not be confirmed in all research studies [24–27].

In summary, despite numerous studies, there is no consensus on the influence of maternal and paternal age at birth on the development or severity of ED symptoms. Given the inconsistencies in the literature, we wanted to explore the hypothesis that the symptom severity, as measured by the well-established Global Severity Index (GSI), is positively associated with age differences between either the mother or the father and the affected patient diagnosed with ED.

## Methods

### Study design and participants

The study cohort consisted of patients from the FamFINED study (FAMily Factors INvolved in Eating Disorders) diagnosed with EDs, who were treated at the Department of Psychosomatic Medicine and Psychotherapy at the University Medical Center Göttingen [18, 28]. The study collected information on diagnoses from all consecutive ED patients treated between the years 1991 and 2017. Study participants were classified as having an ED in accordance with the World Health Organization’s (WHO) 10th International Classification of Diseases and Related Health Problems (ICD-10). Specific diagnoses included anorexia nervosa (ICD-10 codes F50.0 and F50.1) and bulimia nervosa (ICD-10 codes F50.2 and F50.3). Participants were considered eligible for the analysis if they were fluent in the German language and aged 12 years or older at study inclusion. Patients were either adolescent or in adulthood, reflecting the general prevalence of EDs in these age groups [29]. An exclusion criterion was a severe psychiatric disease other than an ED. Participants underwent a systematic diagnostic assessment to classify their ED and determine the diagnosis. Patients were interviewed and psychometrically assessed at baseline by specialists in psychosomatic medicine or licensed clinical psychologists. The patients were asked to provide information on their age, the age of their parents, their living situation, the family constellation, the level of education of the family members, the number of siblings, and the position of the siblings. The highest level of education given was the German Abitur (high school degree). The body mass index was calculated by dividing the body weight in kilograms measured with a calibrated electronic scale under observation, by the height in meters squared. Study participants were asked to answer systematic questions about previous treatments, eating behavior, and their relationship to their own body image to determine their ED symptoms.

Participation in the survey was voluntary, and the ratings were based on self-report in the German language. The subjects and their parents or legal guardians gave their written consent to the scientific analysis and use of their data for research purposes. However, the patients and/or their legal guardians were informed that they had the right to withdraw from the analysis and to terminate participation in the study at any time without giving reasons for this decision. The study was conducted in accordance with the 1964 Declaration of Helsinki. Ethical approval (#32/1/17) for the study was obtained from the Institutional Review Board of the University of Göttingen Medical Center, and the trial was registered in the ClinicalTrials.gov register (#NCT05339165).

### Psychometric assessments

Psychological symptoms were assessed using the German version of the Symptom Checklist-90‑R (SCL-90-R). The self-report SCL-90‑R questionnaire was used to obtain information about the general psychopathological problems of the study participants [30, 31]. The questionnaire consists of 90 items divided into nine scales related to major psychopathological themes, including somatic symptoms, interpersonal sensitivity, obsessive–compulsive behavior, anxiety and depressive symptoms, hostility, phobic symptoms, paranoid tendencies, and psychoticism. Participants were instructed to rate each item over a 7-day observation period on a 5-point scale, with 0 being “not true at all” and 4 being “very true”.

In addition to two other global features measured with the SCL-90‑R (Positive Symptom Total [PST] and Positive Symptom Distress Index [PSDI]), the GSI provides information about the patient’s overall psychopathological distress and overall symptom severity. The GSI score was calculated by adding the values of the items and dividing by 90, excluding missing data. Higher scores on the GSI indicate greater psychopathological distress and symptom severity. The questionnaire has been shown to possess an appropriate level of reliability and validity with a Cronbach’s alpha of 0.97, indicating high internal consistency [32].

### Data analysis

Data from this retrospective study were obtained by electronic transmission of patient records. The Statistical Package for the Social Sciences version 26 (SPSS, IBM, Armonk, NY, USA) was used for statistical analysis. Continuous variables were expressed as means including standard deviations and categorical variables as percentages in accordance with standardized analysis procedures. The study population was divided into groups along the median of the age differences between the patients and both parents. Comparisons between the groups above and below the median were performed separately for each parent using Student’s *t*-test. The median split approach was used, as a control group without an ED diagnosis was not included in the analysis. The chi-squared test was used to determine differences between the two groups for categorical variables such as sex, living situation in the parental home, separated parents, sibling status, living alone, and education level of the patient and the parents. Pearson’s correlations were calculated to determine linear relationships between the overall severity of symptoms, as measured by the GSI score, and the age difference between parent and child. Levene’s test was used to assess the equality of variances between two groups for each parent. Linear regression models were created to examine the effects of the independent variables age difference between mother–patient and father–patient on symptom severity (GSI). The models were adjusted for confounding variables, such as body mass index, living situation, and educational level of the patient, which had been identified as relevant covariates in previous studies [18, 28]. The relative contribution of the predictors was described by the exp(β) coefficient. In the regression models, normal probability plots were routinely used to assess the statistical assumptions of normality and homoscedasticity. Listwise deletion was used to handle missing data. Bonferroni adjustment for multiple testing was not performed due to the pilot nature of this study. Statistical significance was set at a *p*-value of less than 0.05, and the 95% confidence interval (95% CI) was calculated for each variable.

## Results

### Clinical data stratified by median age difference between mother and patient

The total study cohort (*n* = 510) was divided into two groups according to the median age difference between the mother and patient. Patients with a lower age difference to their mothers were significantly older (23.1 ± 7.2 vs. 20.4 ± 5.3 years, *p* < 0.001) and their mothers had a lower education level, defined by a high school degree than those in the group above the median (28.5% vs. 47.0%, *p* < 0.001, Table 1). Patients with younger mothers at delivery were significantly less likely to be living with their parents than patients with older mothers (14.5% vs. 31.1%, *p* < 0.001). In addition, individuals with EDs in the below-median group were assigned a significantly lower sibling position than their counterparts in the above-median group (1.5 ± 0.9 vs. 2.2 ± 1.6, *p* < 0.001). However, no significant differences were found in the study cohort with respect to sex, body mass index, symptom severity assessment (GSI), education level of the patient, living alone, having siblings, and their parents being divorced. Patients with anorexia nervosa (*n* = 252) were evenly distributed along the median for age difference between mother and child (50.2%), whereas this percentage was 62.0% for patients with bulimia nervosa (*n* = 258; *p* = 0.007). Table 1 presents clinical and epidemiological data.

**Table 1:** Characterization of the total study population and comparison of the mother–patient age difference below and above median

	Total study cohort (*n* = 510)	Below median (*n* = 286)	Above median (*n* = 224)	*p* value
Age patient (years)	21.9 ± 6.5	23.1 ± 7.2	20.4 ± 5.3	< 0.001
Sex (female, %)	97.2	97.2	97.3	0.958
Body mass index (kg/m^2^)	18.6 ± 3.0	18.8 ± 3.0	18.4 ± 3.0	0.180
Global Severity Index	1.1 ± 0.63	1.1 ± 0.64	1.1 ± 0.63	0.211
Education level mother (high school degree, %)	36.6	28.5	47.0	< 0.001
Education level father (high school degree, %)	49.1	40.1	60.4	< 0.001
Education level patient (high school degree, %)	52.2	51.0	53.6	0.571
Living alone (%)	42.3	44.7	39.3	0.223
Living in parental home (%)	21.9	14.5	31.1	< 0.001
Parents separated (%)	18.7	20.3	16.7	0.312
Sibling (%)	80.6	81.8	78.9	0.413
Sibling position (order)	1.9 ± 1.3	1.5 ± 0.9	2.2 ± 1.6	< 0.001
Father–patient age difference (years)	31.6 ± 6.0	28.5 ± 4.8	35.5 ± 5.1	< 0.001

### Characterization of the study cohort with regard to the age difference between father and patient

Participants with a father–patient age difference above the median were significantly younger than participants with fathers in the group below the median (20.4 ± 5.3 vs. 23.0 ± 7.0 years, *p* < 0.001, Table 2). Patients with EDs also had younger fathers at their birth when their mothers were below the median age difference (28.5 ± 4.8 vs. 35.5 ± 5.1 years, *p* < 0.001, Table 1). As expected, the age difference mother–patient was higher in the group of patients with older fathers than in patients with younger fathers below the median (32.4 ± 4.3 vs. 25.7 ± 3.8 years, *p* < 0.001, Table 2). The educational level of fathers in the group above the median was significantly higher than that of fathers in the group below the median (60.4% vs. 40.1%, *p* < 0.001, Table 1). A higher proportion of mothers with a high level of education was found in participants with older fathers (46.6% vs. 27.4%, *p* < 0.001, Table 2). Similarly, a higher father–patient age gap was linked to a significantly higher level of education. There were no significant differences between the two groups in terms of gender, body mass index, and living situation, as was the case for the GSI score. However, a significantly lower sibling position was found in the group below the median than in the group above the median (1.5 ± 0.9 vs. 2.3 ± 1.6, *p* < 0.001). Table 2 illustrates the differences between the two groups with regard to the age difference between father and patient.

**Table 2:** Comparison between the two groups with father and patient age differences below and above the median

	Total study cohort (*n* = 491)	Below median (*n* = 260)	Above median (*n* = 231)	*p* value
Age patient (years)	21.9 ± 6.5	23.0 ± 7.0	20.4 ± 5.3	< 0.001
Sex (female, %)	97.1	96.9	97.4	0.746
Body mass index (kg/m^2^)	18.6 ± 3.0	18.7 ± 3.0	18.6 ± 3.0	0.539
Global Severity Index	1.1 ± 0.63	1.2 ± 0.64	1.0 ± 0.61	0.056
Education level mother (high school degree, %)	36.4	27.4	46.6	< 0.001
Education level father (high school degree, %)	48.3	38.3	59.5	< 0.001
Education level patient (high school degree, %)	51.9	50.8	53.2	0.583
Living alone (%)	43.2	43.1	43.2	0.985
Living in parental home (%)	22.0	18.5	25.8	0.057
Parents separated (%)	16.9	19.3	14.2	0.132
Sibling (%)	81.8	80.7	83.1	0.488
Sibling position (order)	1.9 ± 1.3	1.5 ± 0.9	2.3 ± 1.6	< 0.001
Mother–patient age difference (years)	28.8 ± 5.3	25.7 ± 3.8	32.4 ± 4.3	< 0.001

### Correlations between symptom severity and the age difference between parent and child

**Fig. 1:**
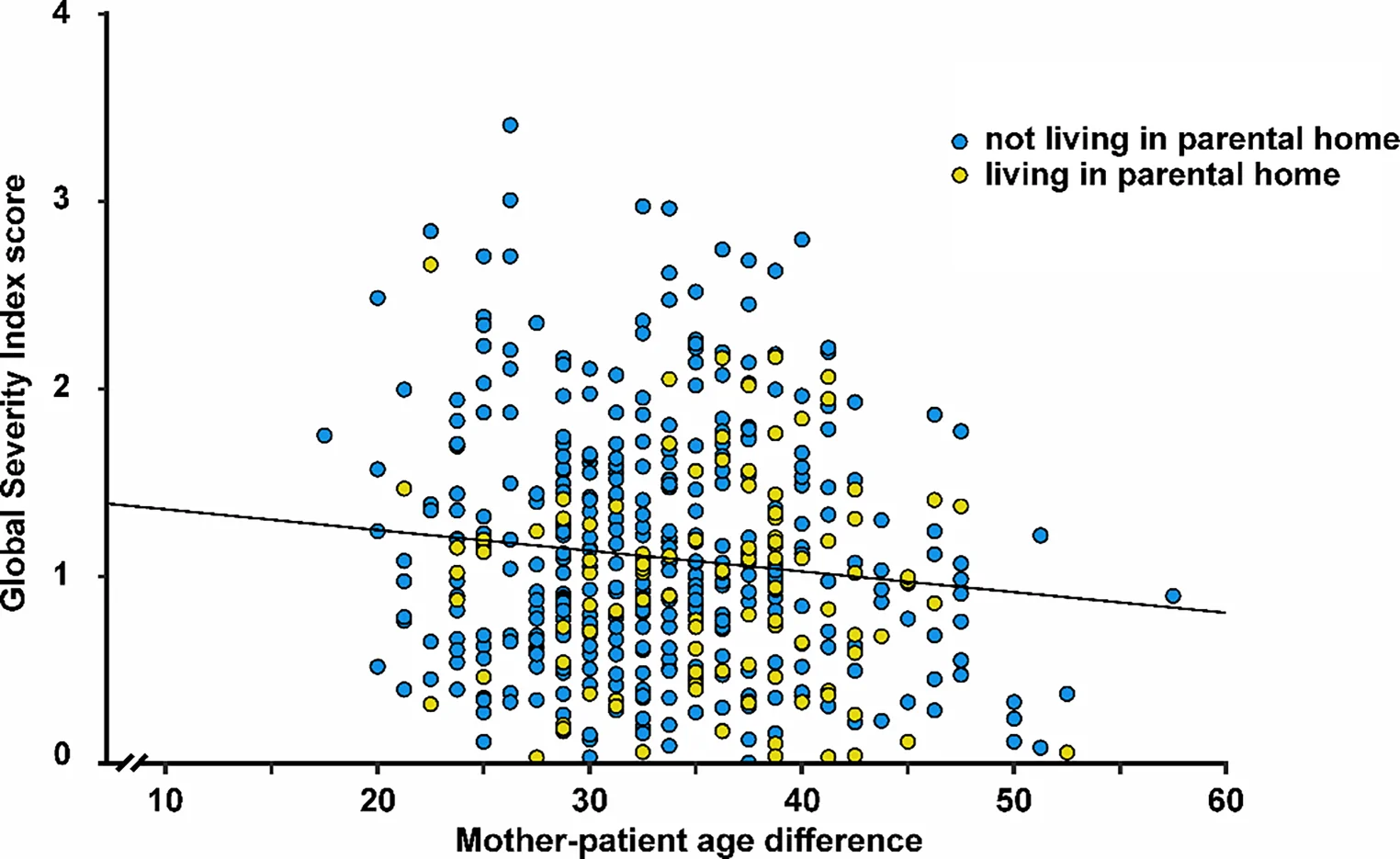
Association between General Severity Index (GSI) score and the age difference between the mother and patient

**Fig. 2:**
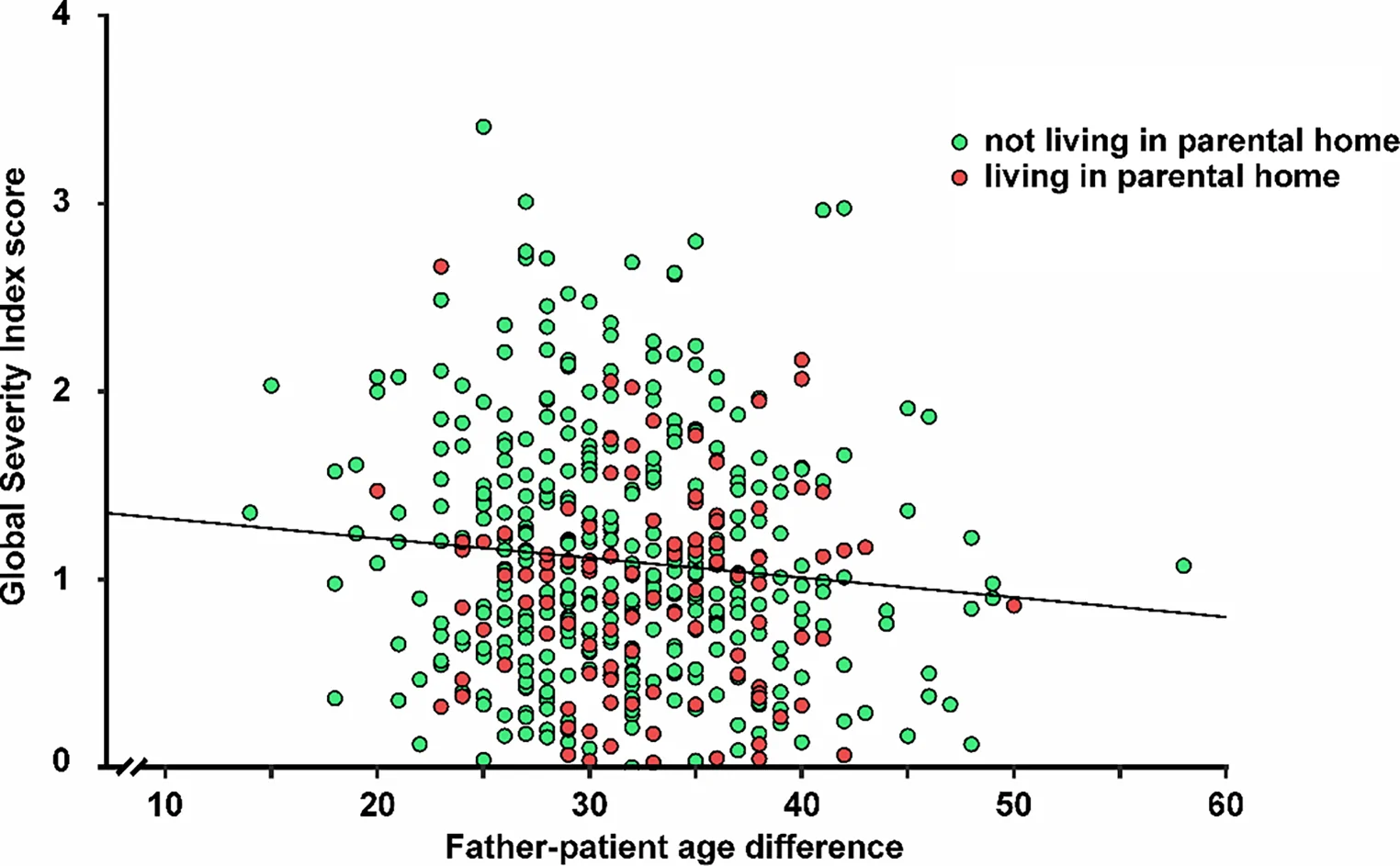
Relationship between General Severity Index (GSI) score and the age difference between the father and patient

Using the Pearson correlation, significant associations were found between the severity of psychological symptoms and the age differences between the patient and the mother and father, respectively. There were similar negative correlations between the GSI score and the age differences between the patient and the mother (r = −0.119, *p* = 0.007, Fig. 1) and the father (r = −0.108, *p* = 0.017, Fig. 2).

### Associations between the general severity index and the parent–patient age differences in multivariate analysis

Binary linear regression models were used to confirm the negative correlation between the GSI score and the age difference between parents and patients in the multivariate analysis (Table 3). Covariates such as body mass index, living situation, and educational level of patients were included in the models to adjust for potential confounding variables. These data confirmed the statistically significant relationship between the GSI score and the age difference between the mother and the patient (exp(β) = −0.091, 95% CI = −0.022 to 0.000, *p* = 0.049; Model 1). The age difference from the father also showed a negative correlation with symptom severity, but did not reach statistical significance (exp(β) = −0.086, 95% CI = −0.018 to 0.000, *p* = 0.063; Model 2).

**Table 3:** The General Severity Index (GSI) score represents the dependent variable in two binary logistic regression models, which were adjusted for body mass index, the living situation, education level of the patient, and additionally expanded for the age difference to the mother (Model 1) or the father (Model 2)

	Exp(β)	Wald	95% confidence interval	*P*-value
*Model 1: (R*^*2*^ *=* *0.047, p* *≤* *0.001)*				
Body mass index (kg/m^2^)	0.089	1.970	[0.000; 0.039]	0.049
Living in parental home (%)	−0.135	−2.801	[−0.351; −0.062]	0.005
Education level of patient (high school degree, %)	−0.130	−2.734	[−0.284; −0.046]	< 0.001
Mother–patient age difference (years)	−0.091	−1.976	[−0.022; 0.000]	0.049
*Model 2: (R*^*2*^ *=* *0.048, p* *≤* *0.001)*				
Body mass index (kg/m^2^)	0.084	1.827	[−0.001; 0.038]	0.068
Living in parental home (%)	−0.158	−3.267	[−0.381; −0.095]	0.001
Education level of patient (high, %)	−0.131	−2.718	[−0.283; −0.046]	0.007
Father–patient age difference (years)	−0.086	−1.864	[−0.018; 0.000]	0.063

## Discussion

The results of the study, which involved 510 outpatients with a diagnosis of EDs, show significant negative correlations between the severity of symptoms and the age gap between the patients and both parents. The association between the GSI score and the age difference between mother and patient remains stable even after controlling for confounding factors such as body mass index, living situation, and educational level of the patient. In a similar regression model with the same confounders, a correlation was also found between symptom severity and the age difference with the father, but this did not reach the statistical significance level. Our results in a large cohort of ED patients support the hypothesis that the age of the parents at the time of the child’s birth plays a role in the development of ED, although the effect sizes are small and may not be clinically relevant.

In the current literature, there is evidence of a greater expression of ED symptoms in the offspring of both younger and older parents [20–23]. According to Wang et al., both younger and older fathers increase the risk of their children developing ED compared to middle-aged fathers [21]. In a nonclinical study sample of 283 female college students, Lofrano-Prado et al. reported a 0.5-time lower likelihood of development of anorexia nervosa in children of mothers over the age of 25 years compared to mothers of a younger age at birth [23]. Fergusson et al. addressed the negative effects of younger parenting on the offspring, who tend to be born into relatively poorly educated and socially disadvantaged families [33]. Furthermore, with increasing age, mothers exert a stronger protective influence on the risk of their children having poorer academic performance and mental health problems than children with parents of average age [34]. Increased maternal age at birth is thought to provide their children with a more stable home environment and is associated with a lower risk of poor academic performance, whereas children with teenage mothers are at higher risk of unfavorable outcomes, including substance abuse and mental health problems later in life [34].

The findings of Allen et al. suggest that children’s perceived satisfaction with their family environment serves as a protective factor against the manifestation of ED symptoms [3]. Our data are consistent with previous research suggesting a significant role of family factors in the development of ED [35, 36]. It also supports the notion that an increased risk of problematic parent–child interactions in mothers who give birth at a younger age may contribute to the development of ED in their offspring [33]. The negative effects of younger parents on symptom severity may also be due to personal factors associated with teenage parenting, as these families tend to have poorer parental monitoring, higher levels of family poverty, and a higher incidence of criminal behavior compared to older parents [37].

Taniguchi observed an increased likelihood of wage loss in mothers who have children at a young age [38]. Research by Ahrén et al. has shown that individuals with the lowest income levels have the highest rates of EDs [39]. Given this information, it is plausible that children of young parents are at increased risk of developing EDs due to the combined effects of unfavorable socioeconomic status and the higher prevalence of EDs among low-income individuals. This hypothesis was also explored by Hooper et al. who described a higher incidence of EDs and teasing in low socioeconomic strata [4]. However, there is also evidence that high social status is a risk factor for the development of anorexia nervosa in children from higher social classes [20]. In addition, a protective effect against bulimia nervosa was found in children aged 10 years with a high socioeconomic status [40].

Kluth et al. observed a significantly higher incidence of ED in adolescent mothers compared to older mothers [41]. A cohort of young parents (mean age 19.8 years) had a 30% higher rate of early EDs compared to the average population [42], suggesting that pathological eating behaviors in young parents may have a direct impact on their offspring. Younger mothers may be more concerned with their appearance which may have influenced the children’s eating behavior (dieting). This assumption is supported by previous studies, such as that of Allen et al. [3], which showed that children of mothers with a history of EDs had more ED symptoms than children from healthy families. These mothers were more concerned about their daughters’ weight than mothers without ED [3]. In a small study population of 11 women, some of their children were found to mimic their mothers’ pathological eating behaviors [43]. In addition, the negative impact of maternal EDs on their children’s eating behavior was confirmed in a study by Reba-Harrelson et al. who concluded that mothers with bulimia nervosa or binge ED reported more eating problems in their children and a restrictive eating style than healthy mothers [44].

Our study has some limitations due to its cross-sectional and retrospective design. The use of established psychometric questionnaires, which ensured anonymity and confidentiality as they were completed in the patients’ home environment, meant that the data on the severity of the patients’ symptoms were based solely on self-report. Relying exclusively on self-reported responses for the analysis can be subject to a variety of response biases, which may potentially inflate the observed correlations due to the tendency of the study participants to answer in a socially desirable manner rather than giving their true opinion. In addition, the study design did not include randomization of study participants or a control group, as only patients diagnosed with ED were studied. Therefore, the study design does not allow any conclusions to be drawn about the causes of ED manifestation or subgroups of ED entities. Although the frequency of ED is higher in girls and women, as in our study population, the heterogeneity of the study cohort could not be guaranteed. Furthermore, dichotomizing a continuous variable via the median split procedure has some statistical disadvantages. Another limitation is that reliable self-reported data on age of symptom onset were not available. During the very long recruitment period, which spanned almost three decades, generational changes may have affected the results, but unfortunately these were not analyzed. The influence of maternal and paternal age differences on male patients needs to be further investigated in future studies. However, our study also has several advantages as it analyzes a well-characterized clinical cohort. The patients were examined and diagnosed by professionally trained mental health specialists. In addition, the large sample size of the cohort studied should be emphasized.

In conclusion, our analysis suggests that there is a significant association between the age difference between the patient and the mother, and probably also the father, and the severity of the child’s eating disorder (ED). The results from our cross-sectional study indicate a higher symptom burden in patients with younger mothers. To better understand the predictive significance of parental age, further studies with a longitudinal design that take potential psychological confounders and different subtypes of ED into account are needed. Given the limited knowledge on this topic, larger studies should be conducted to gather more information on the etiology of EDs to optimize treatment strategies.

## Data Availability

Data will be shared on reasonable request from the corresponding author.
